# Serodiagnosis of *M*. *abscessus* species pulmonary disease using anti-glycopeptidolipid-core IgA antibody

**DOI:** 10.5588/ijtldopen.24.0441

**Published:** 2024-12-01

**Authors:** T. Nii, K. Fujiwara, T. Kobayashi, E. Hagiwara, K. Fukushima, K. Morimoto, K. Tsuyuguchi, T. Ogura, H. Kida

**Affiliations:** ^1^ Department of Respiratory Medicine, NHO Osaka Toneyama Medical Center, Toyonaka, Osaka, Japan;; ^2^ Respiratory Disease Center, Fukujyuji Hospital, Japan Anti-Tuberculosis Association (JATA), Kiyose, Tokyo, Japan;; ^3^ Clinical Research Center, NHO Kinki Chuo Chest Medical Center, Sakai, Osaka, Japan;; ^4^ Department of Respiratory Medicine, Kanagawa Cardiovascular and Respiratory Center, Yokohama, Kanagawa, Japan;; ^5^ Department of Respiratory Medicine and Clinical Immunology, Osaka University Graduate School of Medicine, Suita, Osaka, Japan;; ^6^ Division of Clinical Research, Fukujuji Hospital, JATA, Kiyose, Tokyo, Japan.

**Keywords:** MABS, non-tuberculous mycobacterial pulmonary disease, NTM-PD, Capilia^TM^ MAC

Dear Editor

*Mycobacterium abscessus* species (MABS) ranks as the second most common causal agent of non-tuberculous mycobacterial pulmonary disease (NTM-PD) in Japan, and the number of patients with pulmonary disease caused by this pathogen (MABS-PD) has recently been increasing.^1^ MABS-PD is resistant to antibiotic treatment, and a cure is challenging unless surgical excision is carried out in the early stage of the disease.^2^ Therefore, early diagnosis and treatment are crucial. Glycopeptidolipid (GPL) is a specific cell-wall component found in certain NTM, including *Mycobacterium avium-complex* (MAC) and MABS.**^3^** The IgA antibodies against GPL are specifically elevated in the sera of patients with MAC and MABS-PD, and anti-GPL-core IgA antibody has been developed as a useful serological diagnostic tool for MAC-PD.^4,5^ In addition, serum tests for anti-GPL-core IgA antibodies give a positive result in the early stages of MAC-PD.^6^ However, clinical data on the effectiveness of this antibody test for the diagnosis of MABS-PD are limited.^7,8^ In a previous study, we demonstrated a high positive predictive value (PPV) for the serum anti-GPL-core IgA antibody test in patients who have already met the imaging criteria and had one positive MAC culture.^9^ We also showed that using serum testing for anti-GPL-core IgA antibody tests to diagnose MAC-PD not only saves effort, time, and costs but also enables early diagnosis.

We therefore evaluated whether a similar diagnosis by anti-GPL-core IgA antibody could be made for MABS-PD in a multicenter retrospective study conducted at four tertiary medical institutions in Japan (Osaka Toneyama Medical Center, Osaka; Fukujyuji Hospital, Tokyo; Kinki Chuo Chest Medical Center, Osaka; and Kanagawa Cardiovascular and Respiratory Center, Kanagawa, Japan). A total of 574 patients satisfied the following inclusion criteria: 1) MABS had been detected at least once in sputum culture, 2) serum anti-GPL-core IgA antibodies were measured within 3 months of the first positive culture of MABS, which had been tested between Jan 2006 and December 2022. Of these, we excluded 369 patients in whom MAC was detected before or simultaneously with the first detection of MABS from sputum; the remaining 205 patients were included in our analysis (Figure). Of these 205 patients, serum anti-GPL-core IgA antibodies were detected in 100 patients (48.7%), of whom 73 subsequently met the American Thoracic Society (ATS), European Respiratory Society (ERS), European Society of Clinical Microbiology and Infectious Disease (ESCMID), and Infectious Disease Society of America (IDSA) guideline diagnostic criteria to be diagnosed with ‘definite’ MABS-PD. Serum anti-GPL-core IgA antibodies were not detected in 105 patients (51.2%), of whom 79 were diagnosed with ‘definite’ MABS-PD. In our cohort, 10 patients who were anti-GPL-core IgA antibody-positive and 8 patients who were anti-GPL-core IgA antibody-negative were clinically diagnosed with MABS-PD by an attendant physician and received multidrug therapy. These patients were categorised as ‘probable’ MABS-PD. ‘Definite’ and ‘probable’ MABS-PD accounted for 83.0% of the anti-GPL-core IgA antibody-positive group, indicating a PPV of 83.0% for the anti-GPL-core IgA antibody test among patients with radiologically suspected MABS-PD and a single positive sputum culture test in our cohort (Figure). Subsequently, we evaluated 17 patients in the anti-GPL-core IgA antibody-positive group and 18 in the anti-GPL-core IgA antibody-negative group who had been monitored without multidrug chemotherapy. Notably, cases suspected of being related to contamination were exclusively found in the anti-GPL-core IgA antibody-negative group (2 patients) (Figure). This study was approved by the central institutional review board of Osaka Toneyama Medical Center (TNH-R-2022015).

**Figure.**
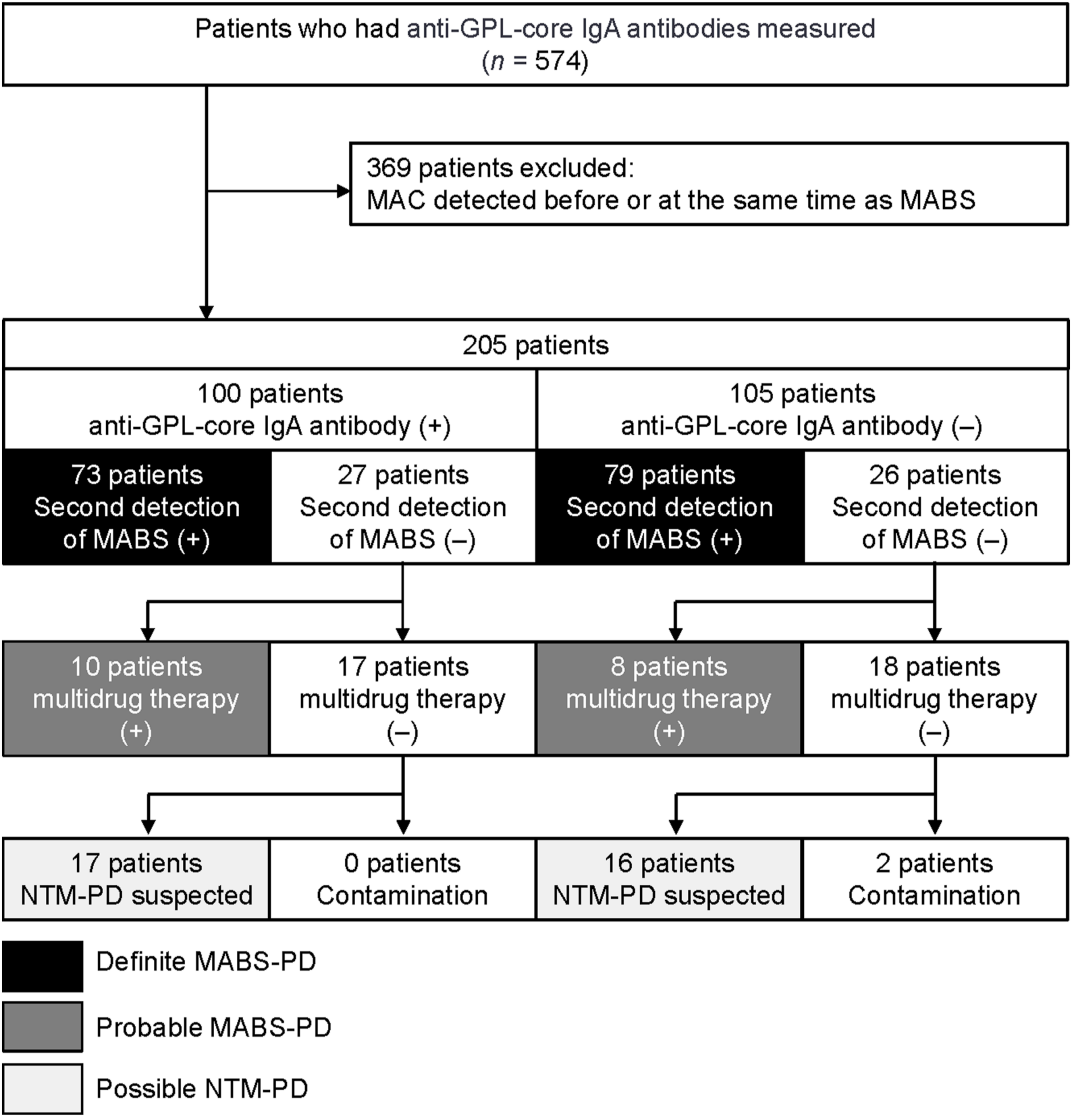
Flowchart of patient categorisation. Based on these numbers, the diagnostic capacity of anti-GPL-core IgA antibody for MABS-PD was as follows: sensitivity 48.8%, specificity 51.4%, negative predictive value 17.1%, and positive predictive value 83%. GPL = glycopeptidolipid; IgA = immunoglobulin A; MAC = *Mycobacterium avium* complex; MABS = *Mycobacterium abscessus* species; + = positive; – = negative; NTM-PD = non-tuberculous mycobacterial pulmonary disease.

Under current international guidelines, at least two positive sputum cultures of the same species of mycobacteria are required to satisfy the microbiologic criteria for diagnosis of NTM-PD.^10^ However, to diagnose the early stage of MAC-PD, patients must repeat the sputum test multiple times to fulfil this criterion or otherwise undergo an invasive bronchoscopy.^11^ In our previous investigation, we illustrated a 95.5% PPV for the serum anti-GPL-core IgA antibody test in patients who had already satisfied imaging criteria and tested positive at least once for MAC in sputum culture. We therefore proposed adding single culture isolation plus anti-GPL-core IgA antibody serum testing as another criterion for the diagnosis of MAC-PD.^9^ In this study, we unexpectedly found that as many as 64% of patients who tested positive for MABS also had a history of MAC being detected in their sputum. In other words, in Japan, more than half of MABS cases present in a manner where it is difficult to determine whether the increase in anti-GPL-core IgA antibody levels is due to MAC or MABS. We also investigated the diagnostic accuracy of anti-GPL-core IgA antibody testing to diagnose MABS-PD. Anti-GPL-core IgA antibody testing had an 83% PPV, a slightly lower value compared with MAC-PD, for the diagnosis of MABS-PD among patients who had already satisfied the imaging criteria and had one positive sputum culture test for MABS. Unlike in the case of MAC-PD, single culture isolation plus anti-GPL-core IgA antibody testing is not suitable for the diagnosis of MABS-PD. Therefore, we need to explore more specific antigens for MABS to enhance the serodiagnosis of MABS-PD. In this study, the sensitivity of anti-GPL-core IgA antibody testing for MABS-PD was 48.8%, which was lower than the reported sensitivity for MAC-PD.^4,5^ Improvements to test sensitivity are crucial.

During the development of the enzyme-linked immune-sorbent assay (ELISA) kit, it became apparent that each patient’s sera reacted differentially to the different serotypes of MAC-derived GPL antigens.^12^ The Capilia^TM^ MAC (Tauns, Shizuoka, Japan), a commercially available ELISA kit for anti-GPL-core IgA antibodies, uses the GPL-core derived from the cell wall of a strain of *M. avium* serotype 4. However, the GPLs of MABS are different from those of MAC.^13^ Thus, to diagnose MABS-PD, we may need a specific ELISA kit, which uses MABS-derived GPLs as antigens to improve the sensitivity. Mutations in GPL biosynthesis and transport pathway can affect GPL surface levels, colony morphology, and MABS virulence,^14^ potentially reducing the sensitivity of anti-GPL-core IgA antibody testing to MABS-PD. To address this, developing an ELISA kit using antigens other than GPL is recommended. Because acid-fast bacteria have various immunogenic cell wall substances, intracellular and secreted proteins, and antibodies against these are present in patients, a panel of serum antibody tests using multiple antigens could form a promising approach.^15^

Our study has some limitations. The retrospective design restricted our ability to control blood sampling conditions. Additionally, there are variations in the sensitivity and specificity of anti-GPL-core IgA antibodies across centres, possibly influenced by regional differences in species. Finally, early cases lacked subspecies identification in MABS-PD, preventing the assessment of the potential for anti-GPL-core IgA antibody testing for each subspecies.
